# Cellular and vaccine therapeutic approaches for gliomas

**DOI:** 10.1186/1479-5876-8-100

**Published:** 2010-10-14

**Authors:** Michelle J Hickey, Colin C Malone, Kate L Erickson, Martin R Jadus, Robert M Prins, Linda M Liau, Carol A Kruse

**Affiliations:** 1 The Joan S. Holmes Memorial Biotherapeutics Research Laboratory, Sanford-Burnham Medical Research Institute, 10901 North Torrey Pines Road, La Jolla, CA 92037, USA; 2 Veterans Affair Medical Center, Long Beach, CA 90822, USA; 3 Department of Neurosurgery and Jonsson Comprehensive Cancer Center, David Geffen School of Medicine, University of California, Los Angeles, Los Angeles, CA 90049, USA

## Abstract

Despite new additions to the standard of care therapy for high grade primary malignant brain tumors, the prognosis for patients with this disease is still poor. A small contingent of clinical researchers are focusing their efforts on testing the safety, feasibility and efficacy of experimental active and passive immunotherapy approaches for gliomas and are primarily conducting Phase I and II clinical trials. Few trials have advanced to the Phase III arena. Here we provide an overview of the cellular therapies and vaccine trials currently open for patient accrual obtained from a search of http://www.clinicaltrials.gov. The search was refined with terms that would identify the Phase I, II and III immunotherapy trials open for adult glioma patient accrual in the United States. From the list, those that are currently open for patient accrual are discussed in this review. A variety of adoptive immunotherapy trials using *ex vivo *activated effector cell preparations, cell-based and non-cell-based vaccines, and several combination passive and active immunotherapy approaches are discussed.

## Introduction

The majority of primary tumors of the central nervous system (CNS) are of astrocytic lineage [1]. Glial tumors are typically classified based upon histologic criteria. The World Health Organization (WHO) classification system for primary malignant gliomas in adults has gradings that range from II to IV. The more slowly growing WHO grade II tumors are termed astrocytomas (A), oligodendrogliomas (ODG), or mixed gliomas (MG). WHO grade III tumors are similarly designated but with the word anaplastic preceding the names, i.e., anaplastic astrocytomas (AA), anaplastic oligodendrogliomas (AODG) or mixed anaplastic gliomas (MAG). The most malignant form, a WHO grade IV glioma is termed a glioblastoma or glioblastoma multiforme (GBM). GBMs are diagnosed at a much higher frequency than the lower grade astrocytomas. Recent GBM groupings-- classified as proneural, mesenchymal, neuronal, or classical-- reflect genetic features of the tumor and have prognostic significance [2,3].

Even with new aggressive standard of care upfront radio-chemotherapy (http://www.clinicaltrials.gov, NCT00006353) [4], the overall survival of GBM patients at two years is dismal at 27.2% [5]. Adjuvant experimental therapies to follow surgical resection and radio-chemotherapy are being explored, amongst them passive and active immunotherapies. Comparing our reviews on immunotherapeutic approaches for brain tumors that were published nearly 10 years ago [6,7] to the present, two obvious changes to the field are evident. First, trials employing active immunotherapy now outnumber those involving passive immunotherapy, and second, investigators are more routinely testing various immune approaches with glioma patients before they exhibit tumor recurrence.

**Figure 1:**
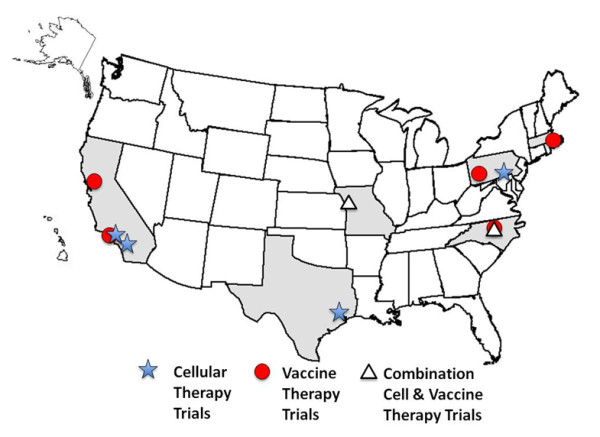
**Map of the United States showing geographical locations of immunotherapy clinical trials discussed in the review**. States shaded in gray have immune therapy clinical trials that are open and currently accruing patients. The city locations of one or more cellular therapy trials are indicated with a blue star, the vaccine therapy trials with a red circle, and the combined cellular and vaccine therapy trials with a white triangle.

We provide a synopsis of the individual active and passive immunotherapy trials and those that use combined active and passive approaches. Three tables summarize the information to include treatment site(s) and lead investigator, an abbreviated trial description, the study phase and estimated enrollment, and indication of whether eligible patients must have recurrent (R), persistent (P) or newly diagnosed (ND) brain tumors at a particular malignant stage (WHO grade). Figure 1 illustrates the geographic distribution of the immunotherapy trials in the United States.

### Cellular Therapy Trials

The adoptive transfer of *ex vivo *activated cytotoxic effector cells to the patient is categorized as a form of passive immunotherapy. The effector cells are administered either systemically or intracranially. If placed intratumorally, the effector cells may be either autologous or allogeneic to the patient. The types of effector cells tested include cytotoxic T lymphocytes (CTL) that are specifically-sensitized to glioma associated antigens (GAA) and exhibit human leukocyte antigen (HLA) restriction [8]. Alternatively, natural killer (NK) or lymphokine activated killer (LAK) cells have been used that are HLA-non-restricted [6,7].

Currently, there are five clinical trials evaluating the safety and effectiveness of cellular therapy approaches (Table 1). At The City of Hope (Duarte, CA), the peripheral blood mononuclear cells (PBMC) from the blood of healthy allogeneic donors are being genetically modified to express a chimeric T cell receptor (TCR) that targets the Interleukin-13 receptor α2 (IL-13Rα2) with a membrane tethered fusion protein known as the IL-13-CD3ζ zetakine (NCT01082926) [9,10]. The zetakine has an E13Y mutation conferring exceptional affinity to the IL-13Rα2 molecule, and reduced affinity to the more commonly expressed IL-13Rα1. Since nearly 80% of high grade primary brain tumors express IL-13Rα2, but normal brain cells do not, the effector cells target the glioma cells [11-14]. Delivery of the gene-modified allogeneic T cells given with aldesleukin (IL-2) for newly-diagnosed patients with WHO grade III or IV brain tumors is by convection enhanced delivery (CED). Concurrent dexamethasone is allowed. The T cell transfectants also express hygromycin phosphotransferase-Herpes simplex virus (HSV) thymidine kinase suicide gene (HyTK) under the control of the cytomegalovirus (CMV) immediate early promoter to provide a method for ablation if graft versus host disease or autoimmunity should occur [9].

**Table 1:** Cellular Therapies for Glioma Patients

Center/Investigator	Therapy/Protocol	Phase - Enrollment	ND, P, R*	WHO Grade***	Clinicaltrials.gov identifier	References
City of Hope, Duarte, CA/B Badie	Allogeneic T Cells modified with chimeric IL-13α2 - TCRζ	I - 10	R, P	III or IV	NCT01082926	Kahlon et al [9]
Baylor College of Medicine, Houston, TX/N Ahmed	Autologous CMV specific CTL genetically modified to target Her2	I/II - 18	ND	IV	NCT01109095	Ahmed et al [18]
Penn State University, Hershey, PA/K Lucas	Allogeneic, CMV specific CTL	I/II - 10	R	IV	NCT00990496	Bao et al [20,72]
UCLA, Los Angeles, CA/L Liau	Alloreactive CTL and IL-2	1 - 15	R	III	NCT01144247	Kruse & Rubinstein [21]
Hoag Cancer Center, Newport Beach, CA/R Dillman	Autologous LAK Cells	II - 80	ND	IV	NCT00814593	Dillman et al [22,73]

* ND, Newly Diagnosed; P, Persistent; R, Recurrent

** World Health Organization (WHO) Grade III: AA, AODG; Grade IV: GBM

Two other clinical trials, one at Baylor College of Medicine (NCT01109095) and another at Penn State University (NCT00990496), evaluate the safety and patient response to intravenous adoptive transfer with autologous or allogeneic CTL, respectively. The CTL target the highly immunogenic human β-herpes cytomegalovirus (hCMV) specific antigens that have been shown to be associated with ~70-90% of glioma cells but not normal brain [15-17]. The CTL for the Baylor trial are additionally gene modified to target HER2, an antigen expressed by nearly 80% of GBMs [18,19]. In this dose escalation trial newly diagnosed GBM patients are treated with one intravenous injection of autologous HER-CMV-CTL. In the Pennsylvania State Phase I/II trial, recurrent or refractory/progressive GBM patients undergo single dose total body irradiation and three days of cyclophosphamide, the intention of which is to eliminate immunosuppressive T regulatory cells (T_reg_) before receiving intravenous infusion of the allogeneic CMV-specific CTL [20].

A dose escalation trial involving intratumoral adoptive transfer of alloreactive CTL (alloCTL) is open for accrual of recurrent glioma patients at the University of California, Los Angeles (UCLA, NCT01144247). After surgical debulking, alloCTL will be placed in the resection cavity. Later alloCTL infusions are delivered through a subgaleal Rickham reservoir/catheter placed at the time of surgery. Patients are treated with 2 alloCTL infusions, 7 days apart to complete 1 cycle. Up to 5 treatment cycles are possible and given every other month. The alloCTL are derived from different donors at each cycle who are allogeneic to the patient. The effector alloCTL are trained *ex vivo *to recognize patient HLA that is highly expressed on the surface of glioma cells but is not present on normal neurons or glia. The trial is predicated upon the results of an earlier pilot study where 3 of 6 recurrent malignant glioma patients demonstrated benefit [21]. One patient survived 40 months, and the remaining two are alive >15 years from the start of immune therapy and entrance into protocol.

At Hoag Cancer Center (Newport Beach, CA), an open, randomized double arm Phase II clinical trial is evaluating the safety of single dose intracavitary autologous LAK cells. This is being compared to Gliadel wafer in newly diagnosed GBM patients (NCT00814593). LAK cells are generated when the patient's PBMC are cultured with high dose recombinant human IL-2 [22].

### Cell Based Vaccine Therapy Trials

Immunization of patients relies upon activation of endogenous immune cells and is categorized as a form of active immunotherapy. In Table 2 (upper half) we list 4 cell-based vaccination trials. Three of the 4 use an autologous dendritic cell (DC) approach to activate the patient's immune system, while 1 uses irradiated autologous whole tumor cells. Another 5 trials (Table 2, lower half) are non-cell based vaccines that employ GAA peptides or complexes that may be combined with immune-potentiating adjuvants. In some cases these therapies will be delivered with other chemotherapeutic agents such as temozolomide (TMZ), or bis-chloroethylnitrosourea (BCNU) or the monoclonal antibody daclizumab which binds to the high affinity alpha subunit (p55 aka CD25) of the IL-2 receptor.

**Table 2:** Vaccine Trials for Glioma Patients

Center/Investigator	Therapy/Protocol	Phase - Enrollment	ND, P, R*	WHO Grade **	Clinicaltrials.gov identifier	References
**Cell-Based Vaccines**						
						
UCLA, Los Angeles, CA/L Liau	Autologous DC + Tumor Lysate	I - 36	ND	III or IV	NCT00068510	Liau et al [46]
Cedars-Sinai, Los Angeles, CA/S Phuphanich	Autologous DC + Synthetic Glioma Peptide	I - 39	R, P	IV	NCT00576641	***
Duke Univ, Durham, NC/D Mitchell	Autologous DC + Brain Tumor Stem Cell-mRNA	I - 50	R	IV	NCT00890032	
Mass General, Boston, MA/W Curry Dana Farber, Boston, MA/P Wen	Autologous Tumor Cells + Irradiated GM-CSF Producing K562 Cells	I - 25	R	III or IV	NCT00694330	

**Non-cell Based Vaccines**						
						
Duke Univ, Durham, NC/D Mitchell	CDX-110 (EGFRviii) Peptide Conjugate + TMZ ± Daclizumab	I/II - 20	ND	IV	NCT00626015	Heimberger et al [74]
Pittsburgh Cancer Center, Pittsburgh, PA/F Lieberman	GAA peptides + PolyICLC	0 - 9	R	II	NCT00874861	Butowski et al [75] ****
Pittsburgh Cancer Center, Pittsburgh, PA/F Lieberman	GAA/TT-peptides + PolyICLC + Montanide ISA-51	0-6	R	II	NCT00795457	
UCSF, San Francisco, CA/A Parsa	Autologous HSPPC-96 vaccine	I/II - 50	R	IV	NCT00293423	Yang & Parsa [76]
UCSF, San Francisco, CA/A Parsa	Autologous HSPPC-96 ± TMZ	II - 63	ND	IV	NCT00905060	

* ND, Newly Diagnosed; P, Persistent; R, Recurrent

** WHO Grade II: A, ODG, MG; Grade III: AA, AODG, MAG; Grade IV: GBM.

*** GAA peptides include: HER-2, TRP-2, gp100, MAGE-1, IL13R alpha, and AIM-2; patients with Brain Stem Glioma are eligible for enrollment

****GAA peptides include: IL-13Ralpha2, Survivin, EphA2 and WT1-derived peptides; GAA/TT includes helper peptide derived from tetanus toxoid

The ongoing Phase I dose-escalation trial at UCLA (NCT00068510) involves DC that are pulsed with autologous tumor cell lysates. The primary endpoint is to evaluate dose limiting toxicity and the maximum tolerated dose of tumor cell lysate pulsed DC in patients with newly diagnosed and recurrent gliomas. Patient response was seen previously when patients received DC pulsed with acid-eluted peptides or tumor lysate administered in combination with chemotherapeutic agents [23,24].

Another variation of the DC vaccine approach is being tested at Cedars-Sinai in Los Angeles (NCT00576641) and is enrolling recurrent WHO grade IV or brain stem gliomas. The approach offers patients with tumor located in unresectable locations an opportunity to receive adjuvant immune therapy. Enrollment into this clinical trial is restricted to patients who are HLA Class I A1 or A2 positive since the synthetic peptides used to pulse the DC are from GAA that display HLA-A1 or -A2 restrictions. Other vaccine trials at Cedars-Sinai (NCT00576537, NCT00576446) using DC pulsed with autologous tumor cell lysates with or without intratumoral Gliadel wafer recently were closed for accrual.

At Duke University (NCT00890032), recurrent GBM patients are treated with autologous DC that are pulsed with mRNA isolated from autologous CD133+ brain tumor stem cells. The method of using mRNA isolated from the patient's own tumor cells to pulse their DC has shown promise in mouse glioma studies, and in an *in vitro *study using human glioma tissue and autologous PBMC [25,26].

Last, at Massachusetts General/Dana Farber Cancer Institute (NCT00694330) a vaccine comprised of irradiated autologous whole tumor cells are given along with K562 cells engineered to produce granulocyte-macrophage colony stimulating factor (GM-CSF), theoretically as a constant source of immune-adjuvant cytokine [27]. Since the K562 erythroleukemic cells, derived from a patient with chronic myelogenous leukemia, express tumor associated antigens such as survivin, hTERT, and Mage-1 in common with gliomas [19,28-31], they also may serve as an additional source of GAA peptides for DC uptake.

### Non-cell-based Vaccine Trials

The lower half of Table 2 summarizes the 5 open non-cell-based vaccine trials currently accruing patients. The first is a Phase I/II trial at Duke University (NCT00626015) that employs a EGFRviii directed-peptide (CDX-110) vaccine that is given intradermally to treat newly diagnosed GBM patients. The EGFRviii variant of EGFR is expressed by nearly a third of glioma specimens [32] therefore the patients enrolled must exhibit positivity for the antigen. The vaccine is administered in conjunction with standard of care TMZ after completion of radio-chemo-therapy. In one arm of the trial patients also receive the anti-IL-2Rα (daclizumab), since T_reg _cells are more sensitive to that antibody compared to the cytotoxic T cell counterpart. Intradermal injections of CDX-110 peptide, or peptide loaded DC has led to increased overall survival in clinical trials without reported autoimmunity [33].

Two Phase 0 clinical trials open at Pittsburgh Cancer Center (NCT00874861, NCT00795457) are evaluating subcutaneous immunization with GAA peptides (IL-13Rα2, Survivin, EphA2 and WT1-derived peptides) and 1 or 2 adjuvants. The first adjuvant is polyinosinic-polycytidylic acid stabilized with polylysine and carboxymethylcellulose (poly-ICLC) that acts as a Toll like receptor 3 agonist and is given intramuscularly 8 times 3 weeks apart. The second adjuvant is Montanide ISA-51, a water-in-oil emulsion that is also given intramuscularly as an immune modulating agent [34]. HLA-A2 positive glioma patients with recurrent grade II tumors are being enrolled.

Two more vaccine trials are open at University of California, San Francisco for recurrent (NCT00293423) or newly diagnosed (NCT00905060) patients with GBM. Enrolled patients are being vaccinated with the heat shock protein peptide complex (HSPPC)-96 with or without concurrent TMZ therapy. Heat shock proteins (HSP) are highly conserved proteins that are transiently expressed during cell stress. They function as molecular chaperones and in the proper folding, assembly, and transport of nascent peptides, and in the degradation of misfolded peptides. Some HSP are highly upregulated on brain tumor cells [35,36]. Interestingly, the gp-96 HSP non-covalently binds to tumor antigens present in the patient's own tumor forming an immunogenic complex that is capable of activating CTL, but neither the gp-96, nor the tumor antigen is immunogenic on its own [37,38].

### Combination Cellular and Vaccine Immunotherapy Trials

Four trials have complex treatment strategies that employ combined active and passive approaches for patients with brain tumors (Table 3). Three currently open clinical trials at Duke University (NCT00639639, NCT00693095, NCT00627224) employ either intradermal vaccination with CMV-specific DCs or CMV-specific autologous lymphocyte transfer (ALT), or both, for newly diagnosed GBM patients. Adoptively transferred CMV-specific CTL reconstitute the hematopoietic system following TMZ-induced lymphopenia that selectively depletes T_reg _cells, and CMV-specific CTL.

**Table 3:** Combined Active and Passive Immunotherapies for Glioma Patients

Center/Investigator	Therapy/Protocol	Phase/Enrollment Number	ND, P, R*	WHO Grade**	Clinicaltrials.gov identifier	References
Duke Univ, Durham, NC/D Mitchell	CMV-DCs ± CMV-ALT + TMZ ± Skin site preparation (unpulsed DC or tetanus toxoid)	I/II - 16	ND	IV	NCT00639639	Mitchell et al [16,77]
Duke Univ, Durham, NC/D Mitchell	CMV-ALT ± CMV-DCs + RT + TMZ (intratumoral CMV-DC upon recurrence)	I - 12	ND	IV	NCT00693095	Mitchell et al [16,77]
Duke Univ, Durham, NC/D Mitchell	CMV-ALT ± CMV-DC or CMV-DC ± CCR7-DC	I/II - 20	ND	IV	NCT00627224	Mitchell et al [16,77]
St. Lukes Hosp, Kansas City, MO/M Salacz	Autologous Tumor Cells + GM-CSF → iv Activated T Cells + IL-2 (TVAX)	I/II - 10	R	III or IV	NCT01081223	Wood et al [39]

* ND, Newly Diagnosed; P, Persistent; R, Recurrent

** WHO Grade III: AA, AODG; Grade IV: GBM

The first trial (NCT00639639) is randomized into 4 arms that evaluate a) CMV-DCs with CMV-ALT, b) CMV-DC alone, c) radiolabeled CMV-DCs following unpulsed DC administration, and d) radiolabeled CMV-DCs following skin site preparations with tetanus toxin. The CMV-specific DCs are pulsed with the pp65-lysosomal-associated membrane protein (LAMP) mRNA and given 3 times. For CMV-ALT, autologous pp65-specific CTL are given once intravenously. The second trial (NCT00693095) involves patient treatment with CMV-ALT with or without CMV-DCs pulsed with pp65 mRNA. Patients will also receive standard of care radiotherapy and TMZ. Interestingly, patients whose tumor recurs following experimental therapy will be offered a resection of the intracavitary tissue with intracranial placement of radiolabeled CMV-DC. The third trial (NCT00627224) similar to the first has four arms: a) CMV-ALT with CMV-DC, b) CMV-ALT alone, c) radiolabeled CMV-DC, and d) radiolabeled CMV-DC that are pulsed with mRNA for the CC chemokine receptor 7 (CCR7) in an effort to direct the CMV-specific DC to the lymph nodes. Upon recurrence, biopsies will be evaluated for DC or CTL infiltrates, and for pp65-antigen escape.

Finally, an open Phase I/II trial at St. Lukes Hospital (Kansas City, MO) combines active and passive immune strategies in patients with recurrent grade III or IV glioma (NCT01081223). Patients are immunized with irradiated autologous tumor cells and GM-CSF (TVAX). Later, autologous T cells are harvested and expanded *ex vivo*, and then administered intravenously. Pilot clinical trials showed promising results with this approach to expand autologous anti-tumor CTL [39]. A similar strategy was employed in two Phase II trials that are either active but not recruiting (NCT00003185) or closed (NCT00004024) [40-42].

### Perspectives On Current Status Of The Field And Future Directions

Six states have immunotherapy trials open for patient enrollment at present with a strong contingency of investigators conducting immune therapy trials concentrated on the west coast of the United States (Figure 1). Comparing these results to reviews that we published nearly a decade ago [6,7] it appears that the overall number of open trials is encouragingly higher. However, while the number of cellular therapy trials remained the same, the clear trend was towards an increase in the number of vaccine trials. Perhaps the costs and the complex logistics associated with generating effector cells for cellular therapy trials influenced this trend.

Commonly, Phase I dose-escalation studies in standard 3+3 design are conducted to ensure safety at any given dose before randomized studies focusing on a particular dose level are initiated. In small Phase 0 and I trials, some now using creative designs with as few as 6-15 patients per arm (see Tables) where toxicity is the primary concern, the likelihood of variability in treatment outcome, especially when they are receiving different doses, is high. Therefore, the studies are underpowered to make robust correlations between clinical outcomes and the immunologic responses generated. Furthermore, there are challenges in making comparative assessments between individual trials. The patient populations treated must be segregated into uniform groups for data analysis. For valid statistical conclusions to be reached one cannot directly compare the outcomes of two individual trials where in one the patients enrolled have persistent or recurrent tumors, and in the other, only recurrent tumors.

Although promising yet anecdotal results have been documented in brain tumor patients treated with a variety of immunotherapeutic approaches [21,43-46] few have advanced from the Phase I/II experimental stage to Phase III testing, testimony of the small number of groups with a research focus in immunotherapy and the constraints placed on NIH for funding such trials because of the current financial climate. Importantly, data gathered from these pilot studies do highlight certain factors that affect response to therapy such as age, maximal resection or minimal/stable residual disease at the start of vaccine therapy, and concurrent administration of chemotherapeutics [23,24,46-51]. For valid conclusions to be reached timely about the value of these approaches more patient participation will be required. Also, with recent advances in new computer-guided surgical techniques, radiation protocols and chemotherapy agents, replacement of older historical control groups with newer ones will be required. With the introduction of new therapies to standard of care for gliomas (i.e., temozolomide, bevacizumab), immunotherapy trials must engender improved survival and quality of life to become integrated into the standard of care regime [5,52-54].

The number of slots open for patient accrual to the immunotherapy protocols contained in our list of open trials totals 489. Based upon the 2010 CBTRUS estimations that 18,980 patients will be diagnosed with a glioma this year in the United States [1], if all available slots were filled in a year, a highly unlikely event, it still would represent only 2.6% participation by the patients in experimental immune testing. Movement toward Phase III trials is encouragingly on the horizon. The largest clinical trial investigating the use of DC vaccines to treat patients with brain tumors (DCVax^®^-Brain) is sponsored by Northwest Biotherapeutics. Although no longer recruiting patients, there are currently 12 institutions participating in the conduct of the Phase II study that is completing treatment and follow-up of 141 enrolled patients http://clinicaltrials.gov/ct2/show/record/NCT00045968[55]. The patients who were treated on the Phase I clinical trials, from which the Phase II study testing DCVax^®^-Brain is predicated, encouragingly continue to demonstrate delays in disease progression and extensions in overall survival http://www.nwbio.com/clinical_dcvax_brain.php[56]. Also, Celldex Therapeutics http://www.celldextherapeutics.com/[57] has plans to conduct a Phase III trial to test EGFRvIII peptide vaccination if the results of their Phase II multi-institutional trial conducted at sites in 15 states http://clinicaltrials.gov/ct2/show/study/NCT00458601 is successful [58,33]. Interim positive results from a Phase 2b brain cancer study with CDX-110, a non-cell based vaccine using an EGFRviii peptide conjugate, given with TMZ were just presented at the 46th Annual ASCO Meeting http://ir.celldextherapeutics.com/phoenix.zhtml?c=93243&p=irol-newsArticle&ID=1434902&highlight=[59]. In addition, ImmunoCellular Therapeutics, Ltd http://www.imuc.com/[60] reports from a recent Phase I study of ICT-107, a DC-based vaccine targeting multiple GAA, that the median overall survival had not yet been reached in patients at the 26.4 month analysis point, with 12 out of 16 treated newly diagnosed GBM patients alive. The company is planning to initiate a phase II study of this vaccine at 15 clinical sites in the second half of 2010 http://www.tradingmarkets.com/news/stock-alert/avrod_imuc_immunocellular-therapeutics-signs-agreement-with-averion-international-to-conduct-phase-ii-glioblast-1176363.html[61]. Finally, Antigenics, Inc. http://www.antigenics.com[62] is supporting a Phase II multi-center single-arm, open-label study to evaluate response to vaccine treatment with Oncophage. Data from 32 evaluable patients treated at UCSF indicate an overall median survival of 44 weeks after tumor resection was achieved, with ~70% of the evaluable patients surviving >36 weeks, and 41% surviving one year or longer. It is clear that clinical trials that address efficacy have been furthered because of support by the biotechnology sector. However, for certain immune therapy products, especially personalized medicinal products produced for diseases with orphan status where the market is small, accompanying support by the National Institutes of Health will be critical.

Furthermore, standardization of the immunologic monitoring endpoints would also help advance the immunotherapy field. Centralized immunologic monitoring laboratories could offer uniform sample handling and analysis. Common endpoints could more reliably provide better comparisons between the individual protocols. Patient responses to immune treatments are assessed over time in cytotoxicity assays by increases in GAA-specific CTL or GAA tetramer analysis in the patients PBMC. Other measurements have included qPCR or Elispot for T helper 1 cytokines, such as IFN-γ, appearance or increases of phenotypically defined cytotoxic subsets in PBMC upon exposure to relevant target cells, and for vaccines in particular, lymphocytic infiltrates at biopsied vaccination sites or tumor site [63-67]. Since it has been noted that patient response to treatment may not always correlate with certain of these laboratory endpoints [46], better definition in this area is needed. Additionally, immunoresistance and genetic variation following immunotherapy could be monitored to address reasons for nonresponse or recurrence [68].

Adjuvant experimental immune therapies are more likely to be of benefit for treating the smaller number of tumor cells remaining after surgical resection. Tumor resection provides an advantage for immune therapies as it helps to reduce the level of immunosuppressive factors produced and secreted by the tumor cells, such as transforming growth factor-beta (TGF-β) or prostaglandin-E2 [69,70]. When the tumor volume is large immunosuppressive factors can be high locally within the tumor microenvironment, and as well, systemically. Overall, surgical resection will have the effect of reducing the number of tumor infiltrating T_reg _cells or myeloid-derived suppressor cells that also can produce immunosuppressive or T helper (Th) 2 or Th3 cytokines such as IL-10 or TGF-β, respectively [68].

Should the single or combined immune therapy modalities be ineffective, combining active or passive immunotherapy approaches with other gene therapy approaches may come to fruition. For instance, our group is currently exploring the possibility of combining alloCTL cellular therapy, now being tested individually (NCT01144247), with gene therapy employing replication competent retroviral vectors encoding suicide genes (NCT01156584), also now being tested individually [71,72]. The combined approaches may not only prove useful for primary malignant brain tumors http://projectreporter.nih.gov/project_info_description.cfm?aid=7746420&icde=4191938[73], but for tumors metastatic to the brain.

Finally, besides contrast-enhanced magnetic resonance imaging (MRI) scans for following brain tumor patient response to immune therapy, other tests should be factored in with those assessments. It is difficult to differentiate inflammation from tumor progression, as both result in enhancement on scans. Follow-up using this one assessment modality has resulted in premature placement of patients off protocol. New experimental MRI and positron emission tomography (PET) techniques are becoming available to help make these assessments and distinguish between pseudo-progression and true tumor progression [74,75]. If validated, the techniques conceivably could be used in conjunction with other less expensive tests to help provide this information. For example, since tumor cells themselves produce and secrete immunosuppressive factors, such as TGF-β, we suggest that serum measurements of TGF-β may be monitored over time as a measure of tumor burden. Its increase systemically, after surgical resection, could offer an indication of tumor regrowth.

## Conclusions

To refine the searches on clinicaltrials.gov we included the following terms: *glioma and biotherapy or immunotherapy, autologous, allogeneic, and vaccine; *we limited the search to trials enrolling *adult *patients and asked for all *Phase I, II and III *trials in the *United States*. Of the listed trials, we focused on those employing cellular therapy, DC or peptide-based vaccines, or combined approaches. Overall, we are encouraged by the advances this field has seen in the last decade. A welcome precedence, the FDA recently approved PROVENGE^®^, a dendritic cell-based vaccine made by Dendreon Corporation http://www.dendreon.com for metastatic, hormone-refractory prostate cancer [76-78]. We look forward to the time when gathered evidence provides implementation of immunotherapeutic approaches to gliomas not only as standard of care, but as first-in-line treatment options. To timelier advance these possibilities, we propose the formation of immunotherapy consortiums that could provide the administrative and statistical oversight and immunologic endpoint integration needed and encourage cooperation between the small cohorts of investigators working in the immune therapy arena. By doing so, integration of novel cellular and vaccine treatments as part of the treatment armamentarium for glioma patients may soon be realized.

## Abbreviations

(A): astrocytoma; (AA): anaplastic astrocytoma; (alloCTL): alloreactive cytotoxic T lymphocytes; (AODG): anaplastic oligodendroglioma; (ALT): autologous lymphocyte transfer; (BTSC): brain tumor stem cell; (CBTRUS): Central Brain Tumor Registry of the United States; (CD): cytosine deaminase; (CED): convection enhanced delivery; (CMV): cytomegalovirus; (CNS): central nervous system; (CTL): cytotoxic T lymphocytes; (DC): dendritic cells; (GAAs): glioma associated antigens; (GM-CSF): granulocyte-macrophage colony stimulating factor; (GBM): glioblastoma multiforme; (hCMV): human cytomegalovirus; (HLA): human leukocyte antigens; (HSP): heat shock protein; (HSPPC): heat shock protein peptide complex; (HSV): herpes simplex virus; (HyTK): hygromycin phosphotransferase-thymidine kinase; (IFN): interferon; (IL): interleukin; (LAK): lymphokine-activated killer; (LAMP): lysosomal-associated membrane protein; (MRI): magnetic resonance imaging; (MHC): major histocompatibility complex; (MAG): mixed anaplastic glioma aka mixed anaplastic oligoastrocytoma; (MG): mixed glioma aka mixed oligoastrocytoma; (MLR): mixed lymphocyte reaction; (mRNA): messenger ribonucleic acid; (ND): newly diagnosed; (NIH): National Institutes of Health; (NK): natural killer; (ODG): oligodendroglioma; (PBMC): peripheral blood mononuclear cells; (P): persistent; (PCR): polymerase chain reaction; (PET): positron emission tomography; (R): recurrent; (TAA): tumor associated antigens; (TCR): T cell receptor; (TGF): transforming growth factor; (TMZ): temozolamide; (TNF): tumor necrosis factor; (Treg): T regulatory cell; (UCLA): University of California, Los Angeles; (UCSF): University of California, San Francisco; (WHO): World Health Organization.

## Conflicting interests

The authors declare that they have no competing interests.

## Authors' contributions

MJH and CAK conceived, outlined the direction of, and drafted the manuscript. MJH, CCM and KLE refined the search for information, gathered references and generated the tables and figure. MRJ, RMP, LML provided information to shape the manuscript content and discussion. All authors have read and approved the final manuscript.
